# Integrating Multiview Information for Enhanced Deep Learning-Based Acute Dermal Toxicity Prediction

**DOI:** 10.1021/acs.jcim.5c02959

**Published:** 2026-03-06

**Authors:** Wei Lin, Chi Chung Alan Fung

**Affiliations:** † Department of Neuroscience, College of Biomedicine, 53025 City University of Hong Kong, Tat Chee Avenue, Kowloon Tong, Kowloon, Hong Kong 999077, China; ‡ CityU Shenzhen Research Institute, 8 Yuexing First Road, Shenzhen Hi-tech Industrial Park, Nanshan District, Shenzhen, Guangdong 518057, China

## Abstract

Accurate prediction of acute dermal toxicity is vital for the safe and effective development of contact drugs. While numerous deep learning models have been created to replace costly and ethically challenging animal toxicity tests, most approaches overlook the multiview information on molecules. To overcome this limitation, we introduce a novel model named MVIToxNet, which integrates multiview features from both molecular fingerprints and SMILES sequences. To capture the multiview information on SMILES, MVIToxNet incorporates character-level and atom-level features. In addition, byte-pair encoding tokenization is utilized to capture substructural details within molecules, allowing the model to differentiate similar SMILES by assigning distinct tokens to different substructures. Since the data sets in this study are small and imbalanced, we argue that selecting a single model based solely on the best validation performance may not reliably reflect the best generalization for test sets. Therefore, we propose a weighted model averaging approach that combines multiple trained models according to their top-K validation scores into one model, yielding an improved model for inference. Extensive experimental results demonstrate that MVIToxNet significantly outperforms existing baselines in acute dermal toxicity prediction, validating the effectiveness of utilizing multiview features and the weighted model averaging strategy. Furthermore, our proposed methods demonstrate the potential for data-driven model design.

## Graphical abstract

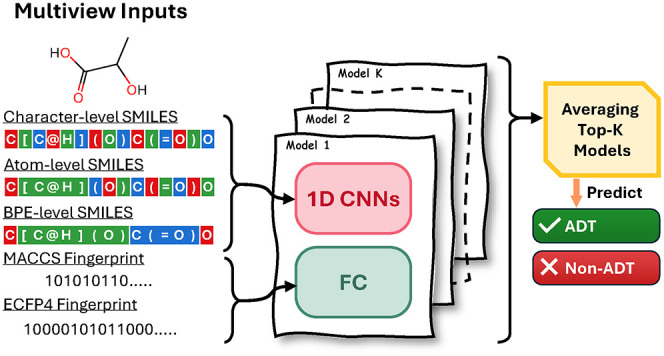

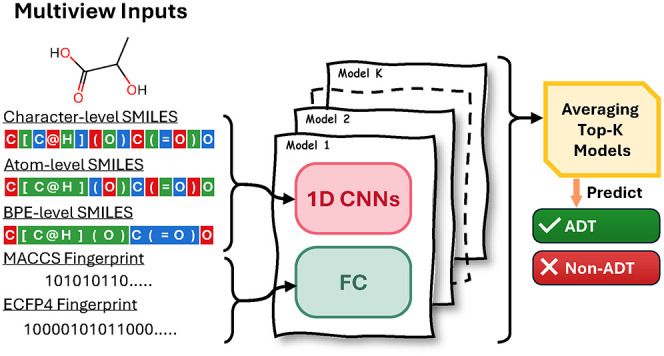

## Introduction

As the body’s largest organ, skin serves a dual role. It guards against external hazards and controls temperature and sensation, yet it is also the primary route by which toxic substances penetrate, rendering acute dermal toxicity (ADT) a critical health risk. ADT describes the adverse effects that appear within 24 h of a single uninterrupted chemical contact with skin.^1^ Reactions can be as mild as redness and swelling or as serious as chemical burns, tissue necrosis, or systemic poisoning if the agent reaches the bloodstream. Consequently, rapid ADT evaluation is a cornerstone of chemical-risk assessment in both workplace safety and regulatory approval.^2,3^ Therefore, accurate prediction of ADT is essential for the safe and effective development of contact drugs.

Conventional toxicity testing heavily relies on animal experiments, which are costly, time-consuming, and raise considerable ethical concerns. Furthermore, the use of animal models is subject to strict ethical and regulatory constraints.^4^ In accordance with the 3Rs principles: reduction, refinement, and replacement of animal testing, there has been increasing adoption of high-throughput in vitro assays and in silico methods to assess potential health risks from environmental agents.^5–7^ As a result, artificial intelligence has become a key tool in recent years for predicting drug toxicity via computational models.^8–15^ For example, RASAR predicts hazards of related compounds using known toxicological data by employing binary fingerprints and the Jaccard distance to measure chemical similarity.^16^ Similarly, STopTox compiles extensive data sets to build machine learning models with fingerprints for the “6-pack” toxicity tests, aiming to evaluate human exposure risks to active ingredients in agrochemicals and related products, including ADT.^17^ However, these traditional computational methods depend heavily on manually engineered features, which restrict their accuracy and ability to generalize. To address these challenges, end-to-end deep learning models have been increasingly developed for toxicity prediction. For instance, GraphADT leverages graph neural networks with structure remapping and multiview graph pooling to predict ADT, demonstrating strong performance in external validation.^18^ Besides, BiModalToxNet and TriModalToxNet, variants of FusionToxNet,^19^ integrate multiple molecular modalities to improve toxicity prediction. Specifically, BiModalToxNet combines molecular fingerprints and molecular images, while TriModalToxNet adds molecular SMILES strings as an additional input.^20^

Despite the excellent predictive abilities of previous models for acute dermal toxicity, several challenges still remain. One key issue is the limited views of input features, which restricts the models’ ability to capture rich information on molecules across different levels. To address this, our proposed MVIToxNet incorporates two widely used types of molecular fingerprints in drug discovery.^21,22^ The first is the MACCS (Molecular ACCess System) keys,^23^ a structural key-based fingerprint predefined by expert knowledge. The second is extended-connectivity fingerprints (ECFPs),^24^ which are circular topological fingerprints capturing the local atomic environments. However, simply utilizing these fingerprints makes the model difficult to distinguish molecules effectively due to the many similar molecules in the data sets. To overcome this, we also exploit multiview information from input SMILES sequences. Specifically, MVIToxNet utilizes character-level and atom-level SMILES representations as inputs. Unlike character-level tokenization, which processes SMILES as individual characters, atom-level tokenization captures chemically meaningful units, such as atoms and their associated properties. However, due to the high structural similarity among many SMILES strings in the data sets, these two tokenization schemes alone are insufficient to fully capture discriminative molecular information. We therefore apply byte-pair encoding (BPE) tokenization,^25^ originally developed in natural language processing to segment sentences into meaningful subunits rather than individual characters. This approach has been successfully adapted to the field of molecules, such as in predicting drug-target interactions^26^ or pretraining molecular models for substructure generation.^27^ In our framework, BPE tokenization splits similar SMILES into distinct substructural tokens, enhancing their differentiation. Additionally, the integration of three complementary SMILES-based features together with two types of molecular fingerprints further enhances the representational capacity of MVIToxNet for acute dermal toxicity prediction.

Another challenge arises from the small size and class imbalance of the data sets used in this work. In such scenarios, selecting the optimal validation model based solely on a single metric may not guarantee the optimal test performance. Therefore, we argue that incorporating models with the top-K performance under a given metric can lead to improved results. To this end, we select several trained models and combine their parameters using a weighted averaging strategy to construct a single ensemble model. This averaged model is then used for inference and consistently delivers performance improvements across diverse evaluation metrics on both the rat and rabbit data sets, demonstrating the effectiveness and robustness of the proposed approach.

In summary, MVIToxNet leverages multiview molecular representations from fingerprints and SMILES sequences to enhance acute dermal toxicity prediction. Notably, we effectively distinguish similar SMILES substructures by employing BPE tokenization. Furthermore, our weighted model averaging approach addresses the limitations of single-metric model selection on the small and imbalanced data sets, enabling better utilization of trained models for improved performance. These proposed methods draw inspiration from the molecular properties used in this work, highlighting the potential for designing models driven by data.

## Materials and Methods

### Data Sets

In this study, the ADT data sets are obtained from previous research,^28^ with compound data compiled from ChemIDplus^29^ and eChemPortal. Only records are explicit LD50 values are included. Compounds are classified according to the Globally Harmonized System (GHS)^30^ as toxic (Categories 1–4, LD50 ≤ 2000 mg/kg) or nontoxic (GHS criteria not met, LD50 > 2000 mg/kg).^1^ To assess model generalizability, two external sets are drawn from the National Institute of Technology and Evaluation (NITE) database.

### Architectures of MVIToxNet

In this study, we present a deep learning model named MVIToxNet, leveraging multiview information of molecules and a weighted model averaging strategy to accurately predict acute dermal toxicity. The architecture of MVIToxNet is exhibited in Figure 1.

**Figure 1:**
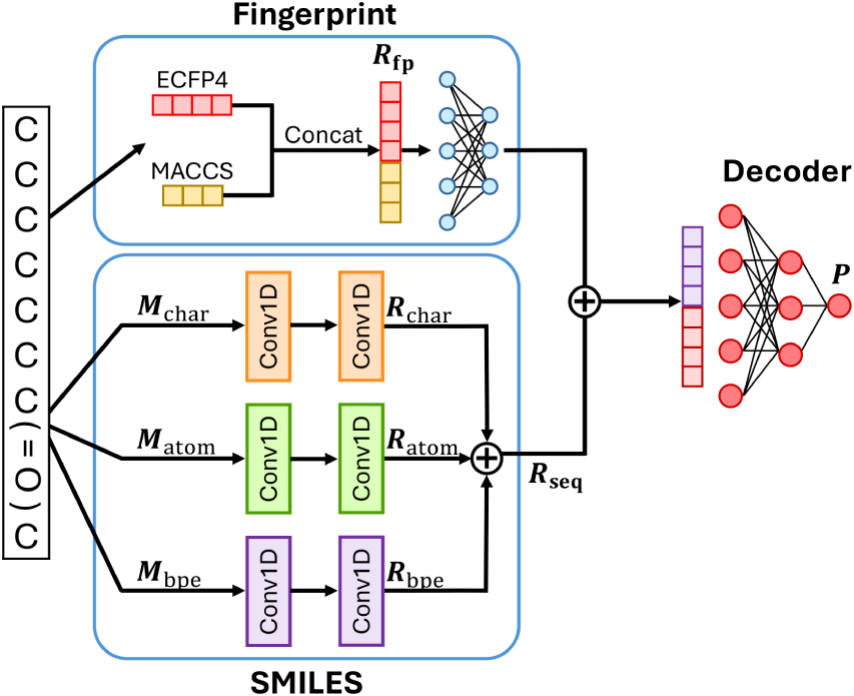
Architecture of MVIToxNet, utilizing multiview information of molecules including character-level, atom-level and BPE-level SMILES strings and two types of fingerprints.

#### Molecular Fingerprint Inputs

For each molecule *M*, the MACCS fingerprint **M**_maccs_ and ECFPs fingerprint **M**_ecfp_ are first concatenated to form **M**_fp_, as shown in eq 1. The concatenated fingerprint **M**_fp_ is then passed through fully connected (FC) layers to produce the fingerprint representation **R**_fp_, as illustrated in eq 2.

1 $$M_{fp}=\mathrm{Concat}\left(M_{maccs},\;M_{ecfp}\right)$$

2 $$R_{fp}=FC\left(M_{fp}\right)$$

#### Sequence-Based Inputs

For the input character-level SMILES **M**_char_, atom-level SMILES **M**_atom_ and SMILES tokenized by BPE **M**_bpe_, we employ three separate 1D convolutional neural networks (CNNs) followed by global max pooling layers to encode **M**_char_, **M**_atom_ and **M**_bpe_ into molecular representation **R**_char_, **R**_atom_ and **R**_bpe_, respectively, as illustrated in eqs 3–5. These two representations are then summed to obtain the sequence representation **R**_seq_, as shown in eq 6.

3 $$R_{char}=\mathrm{MaxPool}\left(\mathrm{CNN}\left(M_{char}\right)\right)$$

4 $$R_{atom}=\mathrm{MaxPool}\left(\mathrm{CNN}\left(M_{atom}\right)\right)$$

5 $$R_{bpe}=\mathrm{MaxPool}\left(\mathrm{CNN}\left(M_{bpe}\right)\right)$$

6 $$R_{seq}=R_{atom}+\alpha \left(R_{char}+R_{bpe}\right)$$

#### Fusion Approach

Finally, the fingerprint representation **R**_fp_ and the sequence representation **R**_seq_ are summed to form the final molecular representation **R**, depicted in eq 7, before going through the decoder to obtain the prediction **P**, as shown in eq 8.

7 $$R=R_{seq}+\beta R_{fp}$$

8 P=FC(R)

#### Definitions of Activation Functions

To better leverage the negative values,^31^ in contrast to ReLU, we utilize different activation functions for different encoders. Specifically, the fingerprint encoder employs the Swish activation function,^32^ as defined in eq 9, while the encoders for SMILES features use the Softplus activation function,^33^ as exhibited in eq 10. Supplementary Figure S1 presents the plots of these two activation functions.

9 Swish(x)=x·σ(x)

10 $$\mathrm{Softplus}\left(x\right)=\log\left(1+e^{x}\right)$$

### Weighted Model Averaging Strategy

In this work, we propose a weighted model averaging strategy, as illustrated in Figure 2. Throughout the training process, we obtain a sequence of trained models {*W*_1_, *W*_2_, ..., *W*_*N*_ }, where *N* represents the total number of training epochs. From this collection, we select the top three models according to their validation AUPRC scores, represented as {*W*_*a*_, *W*_*b*_, *W*_*c*_ }, and assign them corresponding weights {*k*_*a*_, *k*_*b*_, *k*_*c*_ }. These models are combined via weighted model averaging, as defined in eq 11, to produce the final model *W*_Final_. The model with the highest AUPRC is denoted as $W_{{\mathrm{AUPRC}}_{1}}$ . The next two best-performing models from subsequent epochs are denoted as $W_{{\mathrm{AUPRC}}_{2}}$ and $W_{{\mathrm{AUPRC}}_{3}}$ . For the weights, we assign (1 – 2κ) for $W_{{\mathrm{AUPRC}}_{1}}$ but κ for both $W_{{\mathrm{AUPRC}}_{2}}$ and $W_{{\mathrm{AUPRC}}_{3}}$ .

11 $$W_{\mathrm{Final}}=\left(1-2\kappa \right)W_{{\mathrm{AUPRC}}_{1}}+\kappa \left(W_{{\mathrm{AUPRC}}_{2}}+W_{{\mathrm{AUPRC}}_{3}}\right)$$

**Figure 2:**
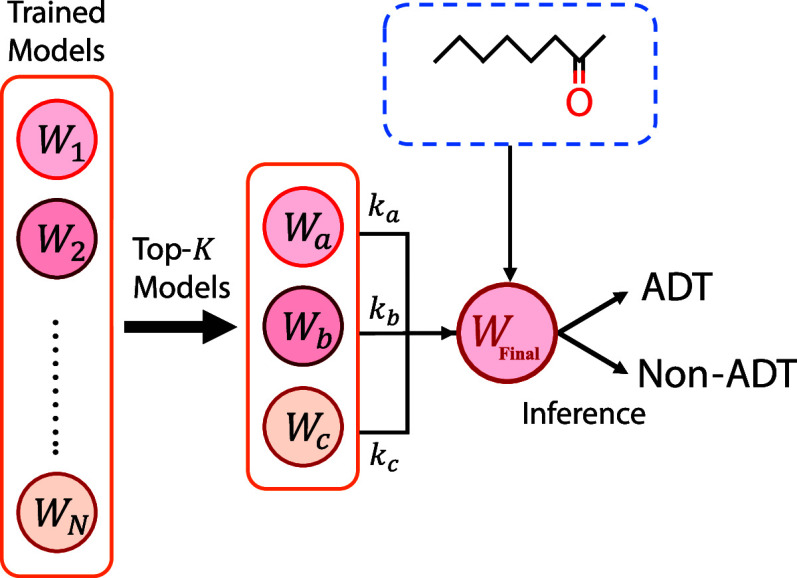
An illustration of the weighted model averaging approach, in which molecular toxicity is predicted by the final averaged model.

### Metrics for Model Evaluation

To comprehensively evaluate our model’s performance against various baselines, we employ multiple metrics in this study, including balanced accuracy (BACC), Area Under the ROC Curve (AUROC) and the Area Under the Precision-Recall Curve (AUPRC), recall, F1-score, and Matthews correlation coefficient (MCC), as shown in eqs 12 [Disp-formula eq17]. The mathematical definitions of these metrics are provided below:

BACC:

12 $$\mathrm{BACC}=\frac{1}{2}\left(\frac{\mathrm{TP}}{\mathrm{TP}+\mathrm{FN}}+\frac{\mathrm{TN}}{\mathrm{TN}+\mathrm{FP}}\right)$$

AUROC:

13 $$\mathrm{AUROC}=\int_{0}^{1}\mathrm{TPR}\left(\mathrm{FPR}\right)\;d\left(\mathrm{FPR}\right)$$

AUPRC:

14 $$\mathrm{AUPRC}=\int_{0}^{1}\mathrm{Precision}\left(\mathrm{Recall}\right)\;d\left(\mathrm{Recall}\right)$$

Recall:

15 $$\mathrm{Recall}=\frac{\mathrm{TP}}{\mathrm{TP}+\mathrm{FN}}$$

F1-score:

16 $$\mathrm{F1‐score}=\frac{2\times \mathrm{TP}}{2\times \mathrm{TP}+\mathrm{FP}+\mathrm{FN}}$$

MCC:

17 $$\mathrm{MCC}=\frac{\mathrm{TP}\times \mathrm{TN}-\mathrm{FP}\times \mathrm{FN}}{\sqrt{\left(\mathrm{TP}+\mathrm{FP}\right)\left(\mathrm{TP}+\mathrm{FN}\right)\left(\mathrm{TN}+\mathrm{FP}\right)\left(\mathrm{TN}+\mathrm{FN}\right)}}$$

where TP, TN, FP, and FN represent true positives, true negatives, false positives, and false negatives, respectively.

## Results and Discussion

### Diagram of Multiview SMILES

For the canonical SMILES “C­[C@H]­(O)­C­(=O)­O”, we adopt three different tokenization methods, namely character-level, atom-level and BPE-level, as exhibited in Figure 3a. In character-level tokenization, the canonical SMILES string is decomposed into individual characters. In atom-level tokenization, the chemically meaningful unit “[C@H]” is treated as a single token, while the remaining elements are split into individual characters. In contrast, BPE tokenization produces four tokens: “C”, “[C@H]­(O)”, “C­(=O)”, and “O”. Here, “[C@H]­(O)” represents a chiral carbon bonded to a hydrogen atom and a hydroxyl group, whereas “C­(=O)” denotes a carbonyl carbon without stereochemical specification. These examples demonstrate that BPE tokenization is also able to capture chemically meaningful substructures from SMILES strings. Moreover, both atom-level and BPE-tokenized SMILES sequences are substantially shorter than character-level sequences, which is consistent with the length statistics reported in Figure 4.

**Figure 3:**
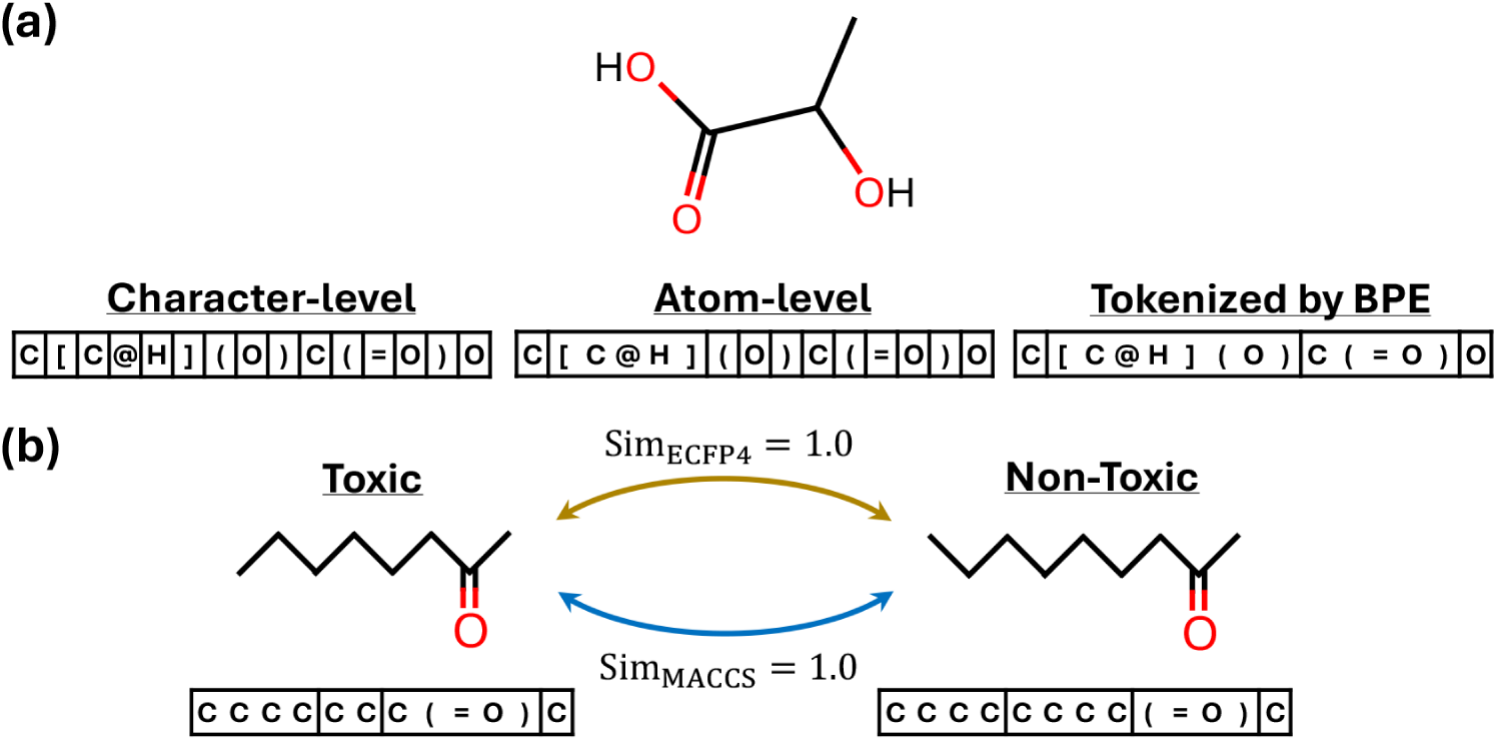
Diagram illustrating multiview information from molecular SMILES: (a) different tokenization methods applied to an example molecule; (b) BPE tokenization of two structurally similar molecules with distinct properties.

**Figure 4:**
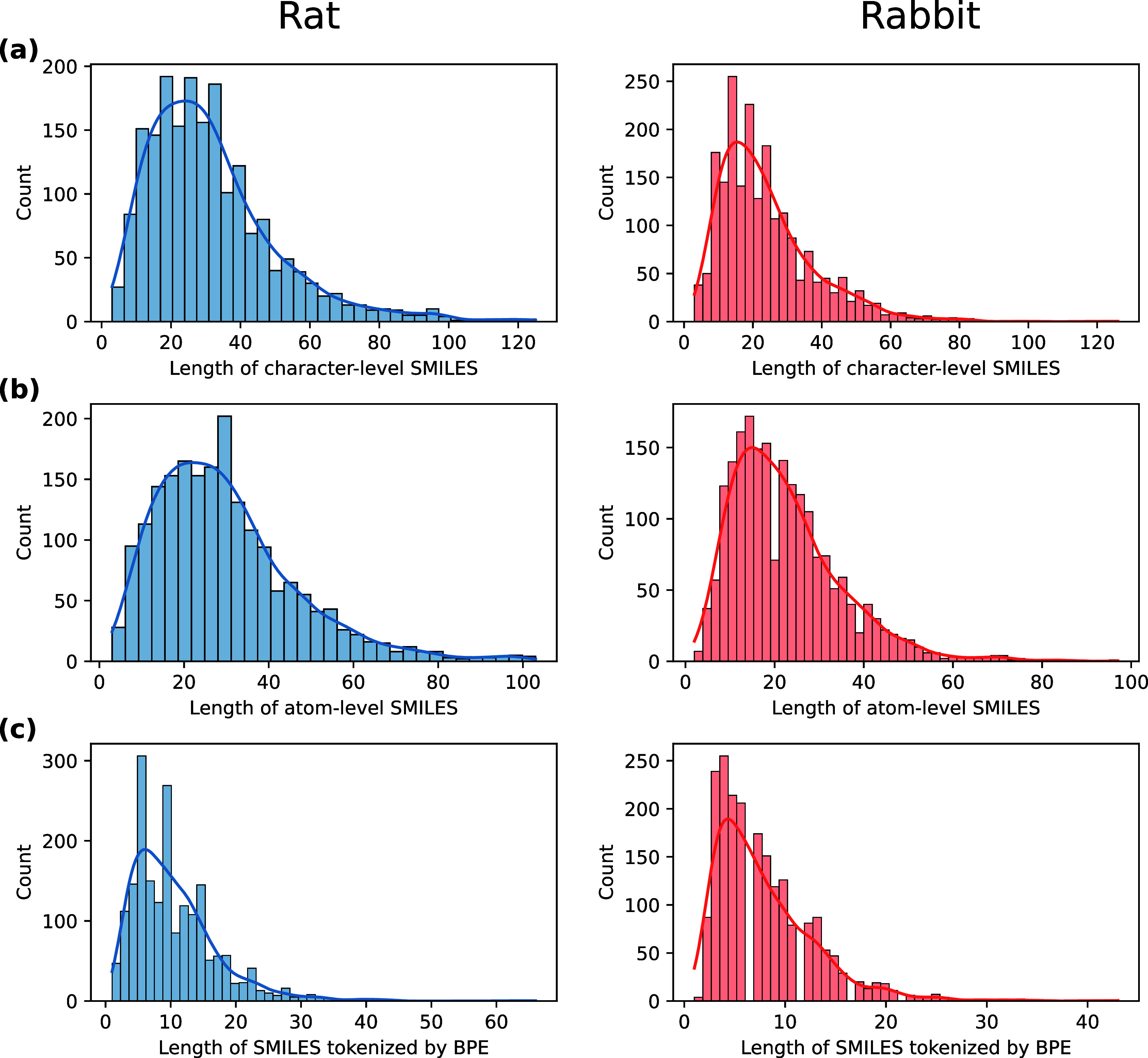
Distribution of SMILES lengths for rabbit and rat data sets: (a) character-level SMILES lengths; (b) atom-level SMILES lengths; (c) BPE-tokenized SMILES lengths.

In Figure 3b, two molecules are structurally similar yet exhibit distinct properties. At first glance, these molecules are challenging to differentiate based on either their SMILES strings or structural images, as they share similar substructures. Moreover, the ECFP4 and MACCS fingerprint similarities between the two molecules are both equal to 1.0, indicating that fingerprint-based representations are insufficient for discriminating between them. To address this challenge, we utilize the BPE tokenization to extract informative molecular substructures. After BPE tokenization, the first molecule is segmented into “CCCC”, “CC”, “C­(=O)”, and “C”, while the second molecule is split into “CCCC”, “(=O)”, and “C” as shown in Figure 3a. These segments correspond to distinct tokens during model training. In contrast, relying solely on character-level or atom-level information could limit the model’s ability to effectively distinguish between such closely related molecules, as their structural difference is confined to a single carbon atom. Besides, we add more example molecules in Supplementary Tables S1 and S2.

### Experimental Settings

In this work, MVIToxNet is implemented with Python 3.9 and PyTorch.^34^ We utilize the BPE vocabulary^25^ trained on 100 million molecules with a size of 498, following the methodology from previous work.^27^ This approach effectively captures frequent substructures while balancing granularity and computational efficiency. As illustrated in Figure 4, the maximum sequence length is set to 80 for character-level SMILES, 60 for atom-level SMILES, and 40 for tokenized SMILES by BPE. Besides, the diameter for ECFP fingerprints is set to 4 (ECFP4), with each fingerprint represented as a 2,048-length vector. The MACCS fingerprint length is 167, including an additional placeholder. All 166 fragment definitions of MACCS are publicly available through RDKit.^35^ We leverage a two-layer FC to deal with the fingerprints. Besides, we use a two-layer CNN with a kernel size of 3 for each branch of the SMILES encoders.

Our experiments are conducted on rat and rabbit data sets using two tree-based models, namely XGBoost,^36^ LightGBM,^37^ and three deep learning models: GraphADT, BiModalToxNet and TriModalToxNet. In addition, we include two pretrained models for comparison. To be specific, embeddings are first extracted and passed through an FC layer for dimensionality reduction, followed by a two-layer CNN. For MolFormer,^38^ we use the MolFormer-XL-both-10pct variant and set the maximum sequence length to 60, reflecting its atom-level vocabulary. For ChemBERTa,^39^ we use the PubChem10M_SMILES_BPE_450k, with a maximum sequence length of 40 due to its BPE-level vocabulary. For training MVIToxNet, we use a batch size of 64 and optimize with the Adam optimizer^40^ at a learning rate of 10^–5^ over 50 epochs. We adopt binary cross-entropy as the loss function for the binary classification task. Data set splits are generated using ten different random seeds (0 through 9). The positive-to-negative sample ratio in the training and validation sets is maintained at 4:1, as detailed in Table 1. External data sets serve as the test sets. All the models are evaluated via 10-fold cross-validation.

**Table 1:** Statistics of Rat and Rabbit Data Sets

Data Set	Rat			Rabbit		
Data Type	Training	Validation	Test	Training	Validation	Test
Total	1343	335	269	1387	347	336
ADT	451	97	73	514	130	124
Non-ADT	892	238	196	873	217	212

### Performance Comparison

We first compare MVIToxNet with several baseline models that do not rely on pretrained features, and the results are summarized in Table 2. MVIToxNet consistently outperforms XGBoost, LightGBM, GraphADT, BiModalToxNet and TriModalToxNet across all evaluation metrics on both the rabbit and rat data sets. To be specific, MVIToxNet achieves improvements of 0.39% in BACC, 2.68% in AUROC, 11.39% in AUPRC, 10.32% in recall, 2.86% in F1-score and 0.19% in MCC compared with the second-best result on the rabbit data set. On the rat data set, MVIToxNet also demonstrates strong performance gains, improving BACC by 0.77%, AUROC by 0.25%, AUPRC by 3.43%, recall by 15.07%, F1-score by 2.51% and MCC by 0.49% over the second-best result. Notably, GraphADT attains the second-best performance on the rabbit data set but performs poorly on the rat data set, whereas TriModalToxNet ranks second on the rat data set but underperforms on the rabbit data set. These phenomena highlight the robustness and superior generalization capability of MVIToxNet across diverse data sets.

**Table 2:** Performance (%) of MVIToxNet Compared with XGBoost, LightGBM, GraphADT, BiModalToxNet, and TriModalToxNet Tested on the Rabbit and Rat Data sets^a^

Data Set	Method	BACC	AUROC	AUPRC	Recall	F1-Score	MCC
Rabbit	XGBoost	62.08	62.08	45.03	50.73	51.63	24.34
	LightGBM	58.6	58.6	42.61	37.02	43.05	18.83
	GraphADT	68.93	74.98	58.12	70.97	61.1	37.49
	BiModalToxNet	63.4	68.43	53.62	66.05	56.57	26.02
	TriModalToxNet	64.49	70.46	55.76	65.4	57.29	28.18
	MVIToxNet	**69.32**	**77.66**	**69.51**	**81.29**	**63.96**	**37.68**
Rat	XGBoost	57.21	57.21	31.32	35.75	36.86	14.78
	LightGBM	55.36	55.36	30.8	19.04	26.77	15.14
	GraphADT	55.69	68.13	44.86	20.0	24.48	16.6
	BiModalToxNet	60.93	67.82	39.92	49.86	44.25	20.62
	TriModalToxNet	61.44	68.78	41.08	52.88	45.21	21.28
	MVIToxNet	**62.21**	**69.03**	**48.29**	**67.95**	**47.72**	**21.77**

a The results represent the average of 10 runs conducted with different random seeds. The best result for each metric is highlighted in bold, while the second-best result is underlined.

We further compare MVIToxNet with two pretrained models, MolFormer and ChemBERTa, as shown in Figure 5. MVIToxNet consistently outperforms both pretrained models across all data sets and evaluation metrics, demonstrating the effectiveness of our proposed approach.

**Figure 5:**
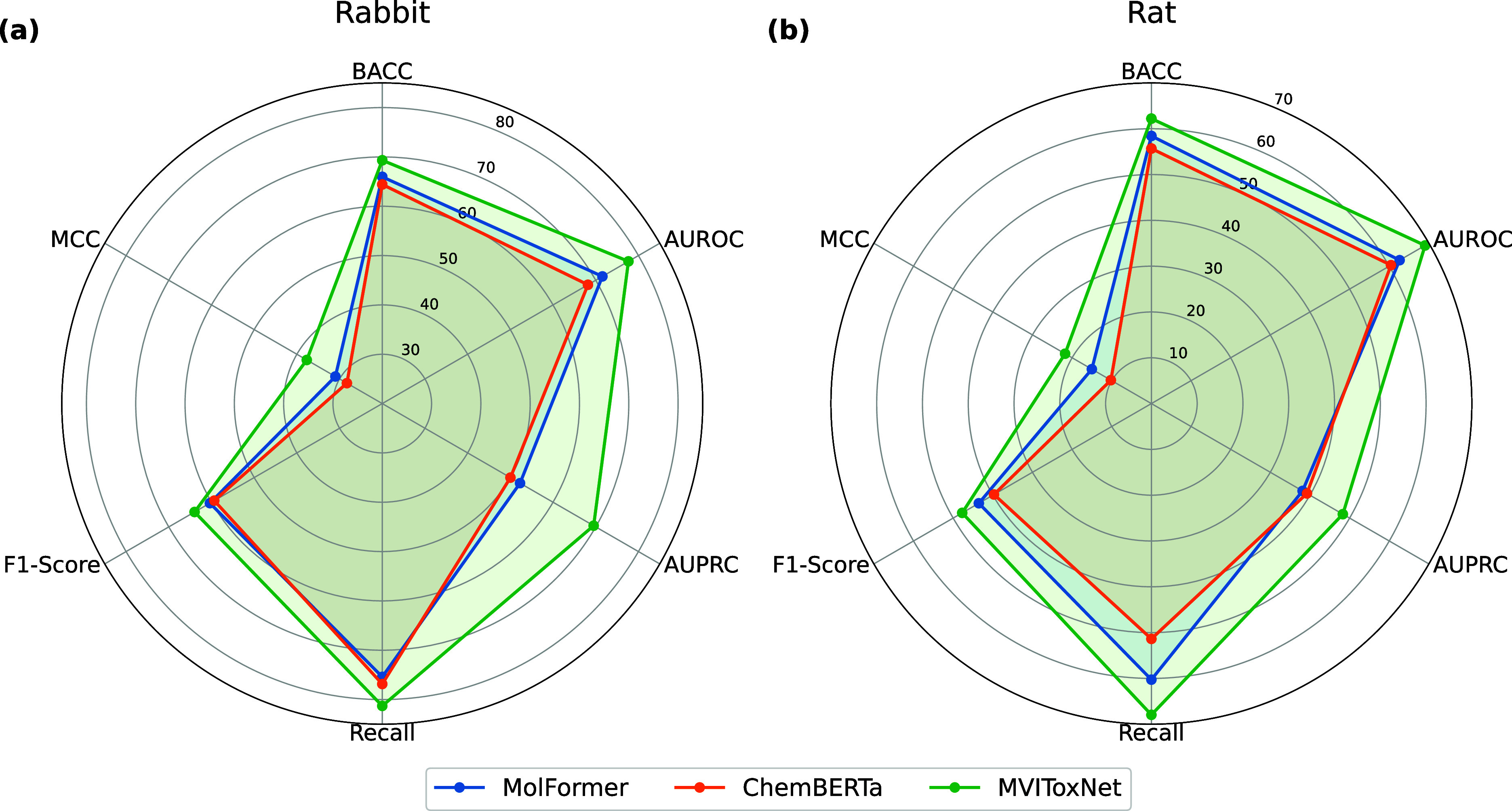
Performance (%) comparison of MVIToxNet with MolFormer and ChemBERTa on (a) rabbit and (b) rat data sets .

### Visualization of MVIToxNet Embeddings

To explore what MVIToxNet learn from the data sets, we apply t-SNE^41^ to visualize the ECFP4 and MACCS fingerprints of molecules as well as the learned embeddings of MVIToxNet on the rat and rabbit external data sets, as presented in Figure 6. We further employ the Davies–Bouldin (DB) index to quantitatively assess clustering separability, where a lower DB index indicates better separation.^42^

**Figure 6:**
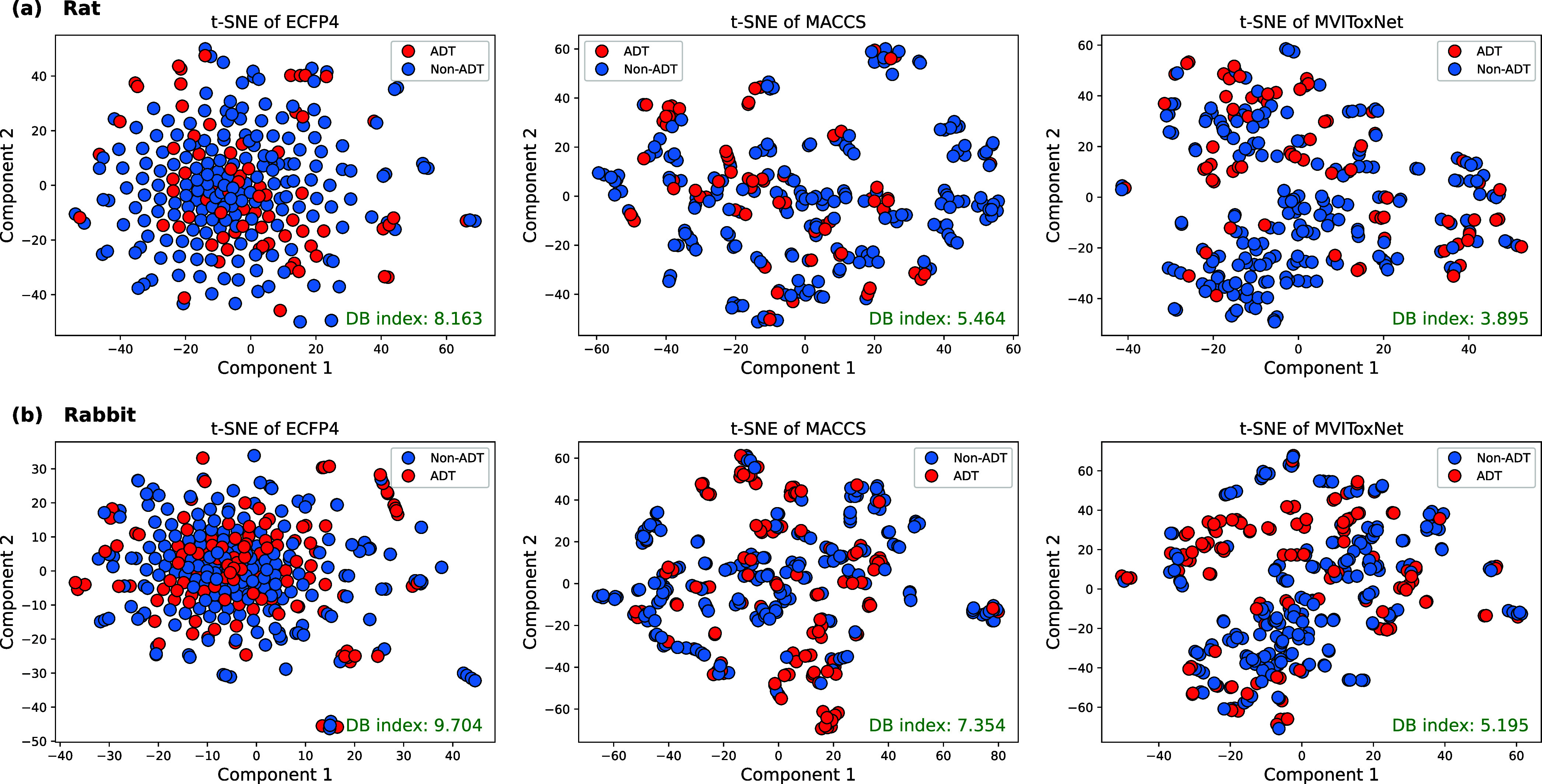
Plots of ECFP4 fingerprints, MACCS fingerprints and embeddings of MVIToxNet on the external sets of the (a) rat and (b) rabbit data sets visualized by t-SNE.

As illustrated in the figure, MVIToxNet embeddings form more distinct clusters and achieve the lowest DB index compared with ECFP4 and MACCS fingerprints, demonstrating that MVIToxNet effectively learns discriminative representations for toxic and nontoxic molecules. Furthermore, the visualizations reveal that many ADT and non-ADT molecules cluster closely in the embedding space, suggesting the presence of similar substructures among certain molecules. This observation is consistent with prior findings^19^ and highlights the need for advanced methods capable of separating structurally similar molecules. Additional visualizations of the rat and rabbit training data sets are provided in Supplementary Figure S2.

### Values of Different Types of Inputs

In this part, we further investigate the impact of different types of molecular inputs. An ablation study of MVIToxNet on the rabbit and rat data sets is presented in Figure 7. Here, “Atom”, “Character”, and “BPE” denote models trained using only atom-level, character-level, and BPE-level SMILES representations, respectively, while “Sequence” and “Fingerprint” correspond to multiview SMILES representations and multiview molecular fingerprints as the inputs.

**Figure 7:**
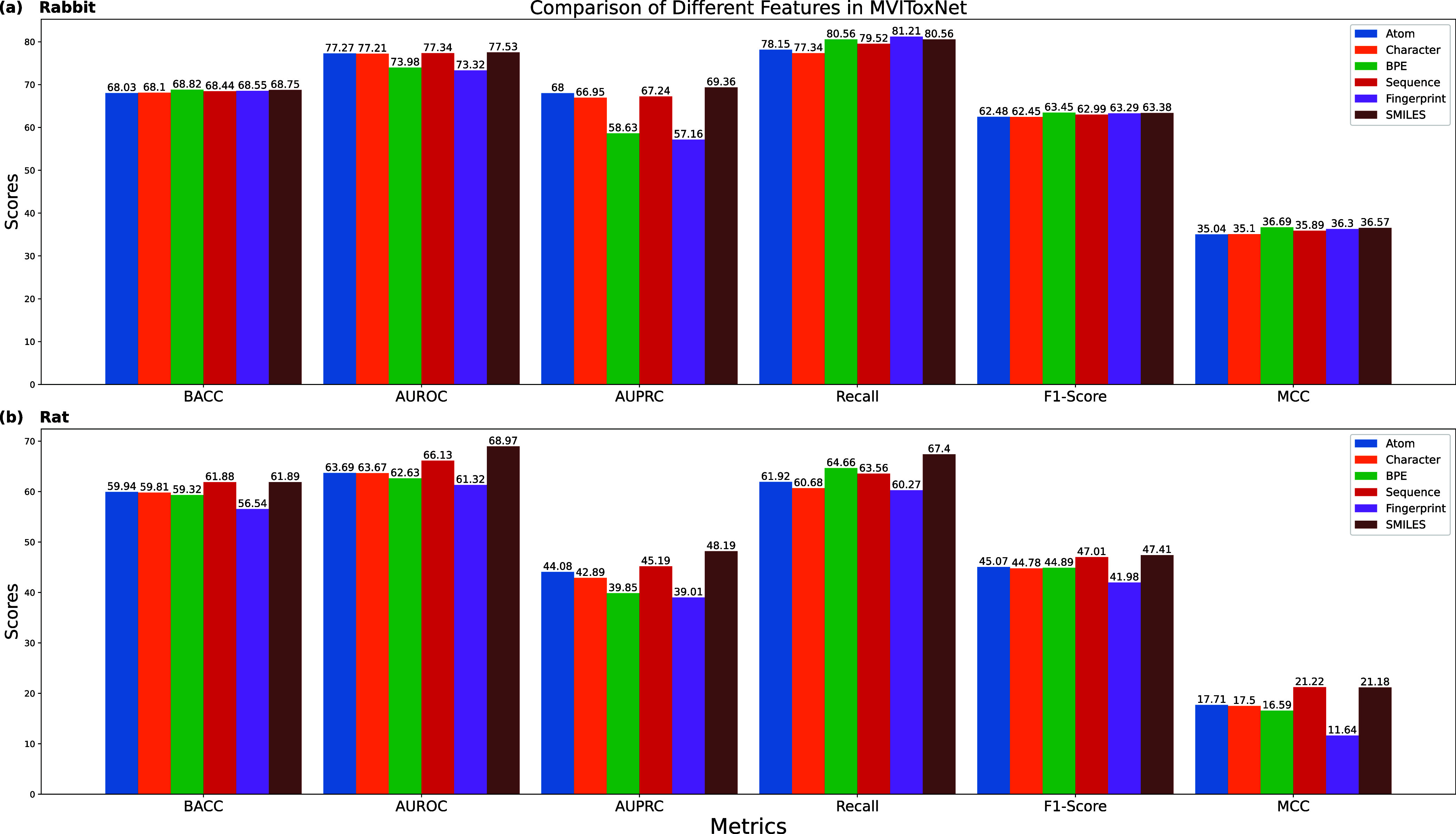
Influence of different features on MVIToxNet’s performance evaluated on the (a) rabbit and (b) rat data sets .

Among single-modality inputs, atom-level SMILES features exhibit the most robust and superior performance across both data sets, with character-level SMILES ranking second. On the rabbit data set, BPE-level features attain the highest scores on metrics including BACC, recall, F1-score, and MCC, but underperform on AUROC and AUPRC compared with the other two SMILES features. Conversely, on the rat data set, BPE-level features generally perform poorly across most metrics, except for recall. This effect may stem from the small data set size and more different tokens generated by BPE tokenization compared with the other two methods. Consequently, we set α = 0.1 in eq 11 to incorporate both character-level and BPE-level SMILES as auxiliary features. As shown in Figure 7, leveraging multiview SMILES representations consistently yields better performance than any single-modality input. In most cases, sequence-based inputs outperform fingerprint-based inputs across all data sets and evaluation metrics, which is consistent with our earlier observation that molecular fingerprints tend to cluster tightly and thus exhibit limited discriminative power when used alone. Accordingly, we set β = 0.1 in eq 7 to treat fingerprints as auxiliary features. Finally, combining SMILES sequence representations with molecular fingerprints leads to further performance gains, underscoring the benefit of integrating multiview molecular representations.

### Effects of Weighted Model Averaging Strategy

To evaluate the effectiveness and generalizability of the proposed strategy, we conduct experiments with κ ∈ [0.0, 0.4] to investigate the impact of κ on the performance of the weighted model averaging strategy. The results are presented in Figure 8, where κ = 0.0 corresponds to the absence of using the model averaging method. For both the rat and rabbit data sets, WMA consistently improves performance across all evaluation metrics, including BACC, AUROC, AUPRC, recall, F1-score, and MCC, demonstrating its effectiveness in enhancing performance on the test sets.

**Figure 8:**
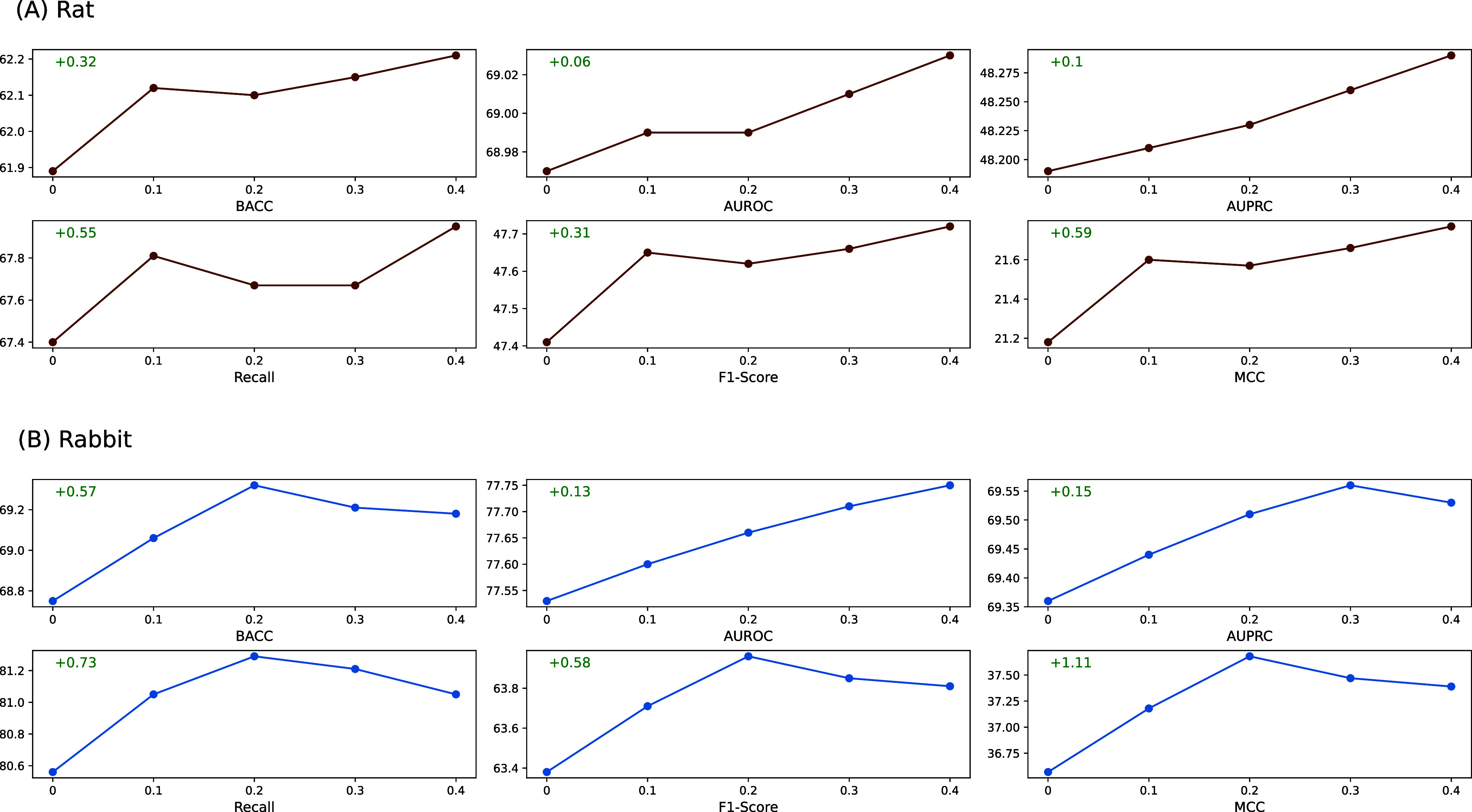
Demonstration of MVIToxNet’s weighted model averaging approach with varying κ on (a) rat and (b) rabbit data sets; the improvements of the combined model compared with the single model are displayed in the figures with κ = 0.4 for the rat data set and κ = 0.2 for the rabbit data set.

On the rat data set, increasing κ leads to consistent performance gains of the combined model over the single model. Consequently, we select κ = 0.4 as the hyperparameter in eq 11 for this study. Compared with the single model, the combined model achieves improvements of 0.32% in BACC, 0.06% in AUROC, 0.1% in AUPRC, 0.55% in recall, 0.31% in F1-score and 0.59% in MCC. For the rabbit data set, the combined model outperforms the single model across all κ values. In particular, it achieves the best results in terms of BACC, AUPRC, recall, F1-score and MCC when κ = 0.2, which is used as the hyperparameter for eq 11 on the rabbit data set. Relative to the single model, the combined model yields improvements of 0.57% in BACC, 0.13% in AUROC, 0.15% in AUPRC, 0.73% in recall, 0.58% in F1-score and 1.11% in MCC.

### Discussion

In the realm of drug discovery, predicting the acute dermal toxicity of candidate molecules is a crucial step following compound selection. Existing approaches predominantly rely on molecular information alone, overlooking potential interactions with protein receptors. Introducing molecule–protein relationships, akin to frameworks used in drug–target interaction studies,^26,43^ offers a promising direction. This reformulation allows for the integration of additional data sets, potentially mitigating limitations arising from data scarcity. Moreover, categorizing toxicity potency into at least three levels could represent a more practical and informative strategy for real-world applications.

Although MVIToxNet has achieved notable success, there remains room for further improvement. Given the strong representational capabilities of pretrained models,^44,45^ a promising direction for further improvement is to incorporate molecular pretrained models based on transformer architectures,^38,39^ which have been widely adopted for molecular modeling tasks.^46–48^ However, fully fine-tuning these models is computationally expensive, while directly using precomputed molecular embeddings often yields suboptimal performance. To address these challenges, low-rank adaptation^49^ offers a parameter-efficient fine-tuning approach that updates only a small subset of model weights, substantially reducing training costs while enhancing model capacity. Furthermore, distinguishing ADT properties that differ due to only a few additional carbon atoms presents a significant challenge. Addressing this issue may require incorporating molecular physicochemical properties to further enrich the model’s representation capacity.^50^

In this study, we also adopt BPE to tokenize molecular SMILES strings. BPE has proven effective in natural language processing, particularly in models such as BERT^51^ and GPT,^52^ by capturing frequent subword patterns. Inspired by this success, BERT-based molecular models^38,39^ have similarly employed BPE to capture meaningful chemical substructures, such as phenyl, carboxyl and aldehyde groups, prior to pretraining. While BERT or GPT typically use large vocabularies, we opt for a smaller BPE vocabulary, aligning with the limited data set sizes in this work to prevent overfitting and maintain efficiency.

The weighted model averaging strategy proposed here shares conceptual similarities with the model soup approach,^53^ which averages models trained under different hyperparameter settings to enhance performance. However, our method differs in that it selects models trained from scratch based on top-K models, rather than varying configurations. Additionally, some work employs the soft voting strategy to aggregate outputs from multiple trained models with different architectures,^54^ which is often at high computational cost. In contrast, our approach maintains efficiency by combining selected models into a single final model. Future work may explore incorporating additional metrics, such as recall and F1-score, or automating the selection of valuable metrics to further refine model selection strategies.

## Conclusion

In this work, we present MVIToxNet, a deep learning-based model designed to integrate multiview features from both molecular fingerprints and SMILES sequences. To effectively capture information from sequence-based inputs, we employ character-level, atom-level SMILES features. Besides, we leverage BPE tokenization, enabling the model to differentiate similar SMILES by assigning unique tokens to different molecular substructures. Experimental results indicate that integrating multiview SMILES features with multiview fingerprints can further enhance the overall performance. Moreover, considering the small size and class imbalance of the data sets, relying on a single evaluation metric may not adequately reflect true model performance. To mitigate this issue, we introduce a weighted model averaging strategy, which aggregates multiple top-K models into a single robust model. This ensemble improves predictive performance on test sets without increasing inference complexity. We conduct extensive experiments on the rat and rabbit data sets. The results demonstrate that MVIToxNet significantly outperforms existing baselines in acute dermal toxicity prediction across all metrics, which underscores the value of multiview feature and model integration and our proposed ensemble approach. The model’s efficiency and effectiveness stem from a data-driven design philosophy at both the molecular and data set levels, exemplifying a data-centric approach to model development.

## Supplementary Material



## Data Availability

The data and codes used in the current study are available at https://github.com/Austin13579/MVIToxNet.

## Notes

Plots of activation functions; examples of compounds sharing identical fingerprints but exhibiting different acute dermal toxicity; t-SNE visualizations of molecules in the training sets (PDF)

## References

[1] OECD Test No. 402: acute dermal toxicity. OECD Guidelines For The Testing Of Chemicals; OECD, 2017.

[2] Lilienblum W., Dekant W., Foth H., Gebel T., Hengstler J., Kahl R., Kramer P.-J., Schweinfurth H., Wollin K.-M. (2008). Alternative methods to safety studies in experimental
animals: role in the risk assessment of chemicals under the new European
Chemicals Legislation (REACH). Arch. Toxicol..

[3] Gu Y., Yu Z., Wang Y., Chen L., Lou C., Yang C., Li W., Liu G., Tang Y. (2024). admetSAR3. 0: a comprehensive platform
for exploration, prediction and optimization of chemical ADMET properties. Nucleic Acids Res..

[4] Van
Norman G. A. (2020). Limitations of animal studies for predicting toxicity
in clinical trials: Part 2: Potential alternatives to the use of animals
in preclinical trials. Basic Transl. Sci..

[5] Krewski D., Andersen M. E., Tyshenko M. G., Krishnan K., Hartung T., Boekelheide K., Wambaugh J. F., Jones D., Whelan M., Thomas R. (2020). Toxicity testing in the 21st century: progress in the
past decade and future perspectives. Arch. Toxicol..

[6] Seal S., Mahale M., García-Ortegón M., Joshi C. K., Hosseini-Gerami L., Beatson A., Greenig M., Shekhar M., Patra A., Weis C. (2025). Machine
Learning for Toxicity Prediction Using Chemical Structures: Pillars
for Success in the Real World. Chem. Res. Toxicol..

[7] Guo W., Liu J., Dong F., Song M., Li Z., Khan M. K. H., Patterson T. A., Hong H. (2023). Review of machine learning and deep
learning models for toxicity prediction. Exp.
Biol. Med..

[8] Tran T. T. V., Surya Wibowo A., Tayara H., Chong K. T. (2023). Artificial intelligence
in drug toxicity prediction: recent advances, challenges, and future
perspectives. J. Chem. Inf. Model..

[9] Braga R. C., Alves V. M., Muratov E. N., Strickland J., Kleinstreuer N., Trospsha A., Andrade C. H. (2017). Pred-skin:
a fast
and reliable web application to assess skin sensitization effect of
chemicals. J. Chem. Inf. Model..

[10] Koutroumpa N.-M., Varsou D.-D., Kolokathis P. D., Antoniou M., Papavasileiou K. D., Papadopoulou E., Papadiamantis A. G., Tsoumanis A., Melagraki G., Velimirovic M. (2025). SbD4Skin by EosCloud:
Integrating multi-view molecular representation for predicting skin
sensitization, irritation, and acute dermal toxicity. Comput. Struct. Biotechnol. J..

[11] Scheufen
Tieghi R., Moreira-Filho J. T., Martin H.-J., Wellnitz J., Otoch M. C., Rath M., Tropsha A., Muratov E. N., Kleinstreuer N. (2024). A Novel Machine Learning Model and a Web Portal for
Predicting the Human Skin Sensitization Effects of Chemical Agents. Toxics.

[12] Huang Z., Lou S., Wang H., Li W., Liu G., Tang Y. (2024). Attentiveskin:
To predict skin corrosion/irritation potentials of chemicals via explainable
machine learning methods. Chem. Res. Toxicol..

[13] Boonsom S., Chamnansil P., Boonseng S., Srisongkram T. (2025). ToxSTK: A
multi-target toxicity assessment utilizing molecular structure and
stacking ensemble learning. Comput. Biol. Med..

[14] Fuadah Y. N., Pramudito M. A., Firdaus L., Vanheusden F. J., Lim K. M. (2024). QSAR Classification
modeling using machine learning
with a consensus-based approach for multivariate chemical hazard end
points. ACS Omega.

[15] Arab, I. ; Barakat, K. ToxTree: descriptor-based machine learning models for both hERG and Nav1. 5 cardiotoxicity liability predictions. arXiv, 2021, 10.48550/arXiv.2112.13467.

[16] Luechtefeld T., Marsh D., Rowlands C., Hartung T. (2018). Machine learning
of
toxicological big data enables read-across structure activity relationships
(RASAR) outperforming animal test reproducibility. Toxicol. Sci..

[17] Borba J. V., Alves V. M., Braga R. C., Korn D. R., Overdahl K., Silva A. C., Hall S. U., Overdahl E., Kleinstreuer N., Strickland J. (2022). STopTox: An in silico alternative to animal
testing for acute systemic and topical toxicity. Environ. Health Perspect..

[18] Ma X., Fu X., Wang T., Zhuo L., Zou Q. (2024). GraphADT: empowering
interpretable predictions of acute dermal toxicity with multi-view
graph pooling and structure remapping. Bioinformatics.

[19] Madheswaran M., Jaganathan K., Shanmugam L. (2025). Deep learning-based multimodal fusion
approach for predicting acute dermal toxicity. J. Chem. Inf. Model..

[20] Weininger D. (1988). SMILES, a
chemical language and information system. 1. Introduction to methodology
and encoding rules. J. Chem. Inf. Comput. Sci..

[21] Mellor C. L., Robinson R. L. M., Benigni R., Ebbrell D., Enoch S. J., Firman J. W., Madden J. C., Pawar G., Yang C., Cronin M. T. D. (2019). Molecular fingerprint-derived
similarity measures for
toxicological read-across: Recommendations for optimal use. Regul. Toxicol. Pharmacol..

[22] Wigh D. S., Goodman J. M., Lapkin A. A. (2022). A review of molecular
representation
in the age of machine learning. Wiley Interdiscip.
Rev.: Comput. Mol. Sci..

[23] Durant J. L., Leland B. A., Henry D. R., Nourse J. G. (2002). Reoptimization of
MDL keys for use in drug discovery. J. Chem.
Inf. Comput. Sci..

[24] Rogers D., Hahn M. (2010). Extended-connectivity fingerprints. J. Chem.
Inf. Model..

[25] Sennrich, R. ; Haddow, B. ; Birch, A. Neural Machine Translation of Rare Words with Subword Units. In Proceedings of the 54th Annual Meeting of the Association for Computational Linguistics; Association for Computational Linguistics: Berlin, Germany, 2016; pp. 1715–1725.

[26] Huang K., Xiao C., Glass L. M., Sun J. (2021). MolTrans: molecular
interaction transformer for drug–target interaction prediction. Bioinformatics.

[27] Chitsaz, K. ; Balaji, R. ; Fournier, Q. ; Bhatt, N. P. ; Chandar, S. NovoMolGen: Rethinking Molecular Language Model Pretraining. arXiv, 2025, 10.48550/arXiv.2508.13408.

[28] Lou S., Yu Z., Huang Z., Wang H., Pan F., Li W., Liu G., Tang Y. (2024). In silico prediction of chemical acute dermal toxicity
using explainable machine learning methods. Chem. Res. Toxicol..

[29] Tomasulo P. (2002). ChemIDplus-super
source for chemical and drug information. Med.
Ref. Serv. Q..

[30] CHEMICALS, L. O. Globally harmonized system of classification and labelling of chemicals (GHS), 2002.

[31] Lin W., Piao Z., Fung C. C. A. (2025). Delving into Unsupervised Hebbian
Learning from Artificial Intelligence Perspectives. Mach. Learn. Knowl. Extr..

[32] Ramachandran, P. ; Zoph, B. ; Le, Q. V. Searching for activation functions. arXiv, 2017, 10.48550/arXiv.1710.05941.

[33] Glorot, X. ; Bordes, A. ; Bengio, Y. Deep sparse rectifier neural networks Proceedings Of The Fourteenth International Conference On Artificial Intelligence And Statistics PMLR 2011 15 315–323

[34] Paszke, A. ; Gross, S. ; Massa, F. ; Lerer, A. ; Bradbury, J. ; Chanan, G. ; Killeen, T. ; Lin, Z. ; Gimelshein, N. ; Antiga, L. , . Pytorch: An imperative style, high-performance deep learning library. In Advances In Neural Information Processing Systems; Curran Associates, Inc., 2019, Vol. 32.

[35] Landrum, G. , RDKit: open-source cheminformatics. https://github.com/rdkit/rdkit, 2006.

[36] Chen, T. ; Guestrin, C. Xgboost: A scalable tree boosting system Proceedings Of The 22nd ACM SIGKDD International Conference On Knowledge Discovery And Data Mining Association for Computing Machinery 2016 785–794

[37] Ke, G. ; Meng, Q. ; Finley, T. ; Wang, T. ; Chen, W. ; Ma, W. ; Ye, Q. ; Liu, T.-Y. Lightgbm: A highly efficient gradient boosting decision tree. In Advances In Neural Information Processing Systems; Curran Associates, Inc., 2017, Vol. 30.

[38] Ross J., Belgodere B., Chenthamarakshan V., Padhi I., Mroueh Y., Das P. (2022). Large-scale chemical language representations capture molecular structure
and properties. Nat. Mach. Intell..

[39] Chithrananda, S. ; Grand, G. ; Ramsundar, B. ChemBERTa: large-scale self-supervised pretraining for molecular property prediction. arXiv, 2020, 10.48550/arXiv.2010.09885.

[40] Kingma, D. P. ; Ba, J. Adam: A method for stochastic optimization. arXiv, 2014, 10.48550/arXiv.1412.6980.

[41] van
der Maaten L., Hinton G. (2008). Visualizing data using t-SNE. J. Mach. Learn. Res..

[42] Fang Y., Zhang Q., Zhang N., Chen Z., Zhuang X., Shao X., Fan X., Chen H. (2023). Knowledge graph-enhanced
molecular contrastive learning with functional prompt. Nat. Mach. Intell..

[43] Lin W., Fung C. C. A. (2025). Utilizing Data Imbalance to Enhance Compound–Protein
Interaction Prediction Models. Adv. Intell.
Syst..

[44] Lin W., Fung C. C. A. (2024). Rethinking the Masking Strategy for Pretraining Molecular
Graphs from a Data-Centric View. ACS Omega.

[45] Fang X., Liu L., Lei J., He D., Zhang S., Zhou J., Wang F., Wu H., Wang H. (2022). Geometry-enhanced molecular
representation learning for property prediction. Nat. Mach. Intell..

[46] Khambhawala A., Lee C. H., Kwon J. S.-I. (2025). Accelerating
Drug Discovery with
Hybrid Physiologically Based Pharmacokinetic (PBPK) Models: A Transformer-Based
Approach for Pharmacokinetic Predictions. Ind.
Eng. Chem. Res..

[47] Madani M., Lacivita V., Shin Y., Tarakanova A. (2025). Accelerating
materials property prediction via a hybrid Transformer Graph framework
that leverages four body interactions. Npj Comput.
Mater..

[48] Khambhawala A., Lee C. H., Pahari S., Kwon J. S.-I. (2025). Minimizing late-stage
failure in drug development with transformer models: Enhancing drug
screening and pharmacokinetic predictions. Chem.
Eng. J..

[49] Hu E. J., Shen Y., Wallis P., Allen-Zhu Z., Li Y., Wang S., Wang L., Chen W. (2022). Lora:
Low-rank adaptation of large language models. ICLR.

[50] Pahari S., Lee C. H., Sitapure N., Kwon J. S.-I. (2025). Predicting both
thermodynamic and kinetic properties of crystallizing molecules via
transformer-based language model. AIChE J..

[51] Devlin, J. ; Chang, M.-W. ; Lee, K. ; Toutanova, K. Bert: Pre-training of deep bidirectional transformers for language understanding Proceedings Of The 2019 Conference Of The North American Chapter Of The Association For Computational Linguistics: human Language Technologies Association for Computational Linguistics 2019 4171–4186

[52] Brown, T. ; Mann, B. ; Ryder, N. ; Subbiah, M. ; Kaplan, J. D. ; Dhariwal, P. ; Neelakantan, A. ; Shyam, P. ; Sastry, G. ; Askell, A. Language models are few-shot learners Advances In Neural Information Processing Systems Curran Associates, Inc. 2020 33 1877–1901

[53] Wortsman, M. ; Ilharco, G. ; Gadre, S. Y. ; Roelofs, R. ; Gontijo-Lopes, R. ; Morcos, A. S. ; Namkoong, H. ; Farhadi, A. ; Carmon, Y. ; Kornblith, S. Model soups: averaging weights of multiple fine-tuned models improves accuracy without increasing inference time International conference on machine learning PMLR 2022 23965–23998

[54] Yao L., Wang F., Xie P., Guan J., Zhao Z., He X., Liu X., Chiang Y.-C., Lee T.-Y. (2025). StackPIP: An Effective
Computational Framework for Accurate and Balanced Identification of
Proinflammatory Peptides. J. Chem. Inf. Model..

