# Supplementary material for: Integrating Multiview Information for Enhanced Deep Learning-Based Acute Dermal Toxicity Prediction
Source: J Chem Inf Model. 2026 Mar 6;66(6):3049–58. doi: 10.1021/acs.jcim.5c02959 (PMC13014460; doi:10.1021/acs.jcim.5c02959)
Supplement: Supplementary file 1 [file ci5c02959_si_001.pdf]

Supporting Information: Integrating Multiview Information for Enhanced Deep Learning-Based Acute Dermal Toxicity Prediction

Wei Lin[*,†] and Chi Chung Alan Fung[*,†,‡]

†*Department of Neuroscience, College of Biomedicine, City University of Hong Kong, Tat Chee Avenue, Kowloon Tong, Kowloon, Hong Kong, China*

‡*CityU Shenzhen Research Institute, 8 Yuexing 1st Road, Shenzhen Hi-tech Industrial Park, Nanshan District, Shenzhen, Guangdong, China*

E-mail: wlin44-c@my.cityu.edu.hk; alan.fung@cityu.edu.hk

S1. Visualization of Two Activation Functions

We visualize the differing behaviors of two different activation functions, namely Swish[1] and Softplus,[2] near zero, which is shown in Supplementary Figure S1.

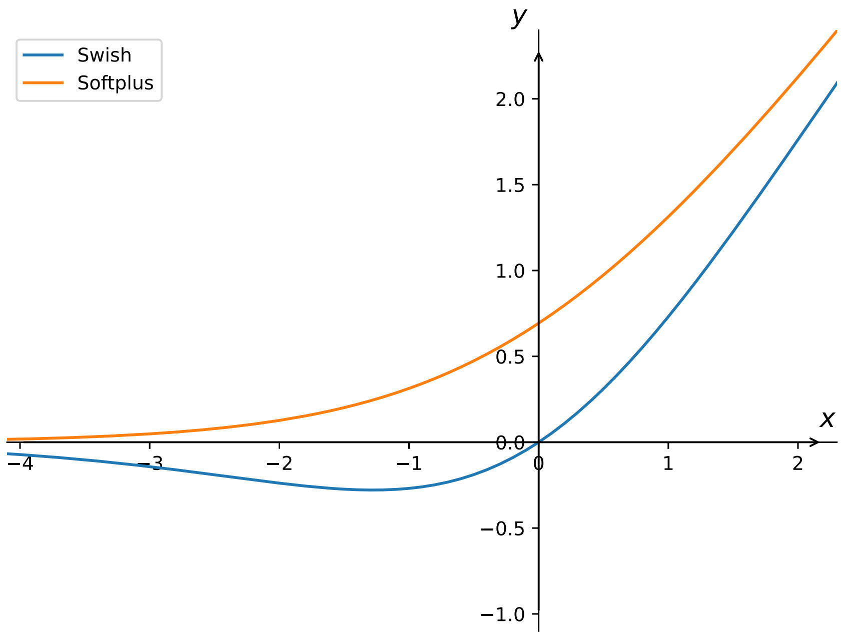

Figure S1: Visualization of Swish and Softplus activation functions around zero.

S2. Examples of Structurally Similar Molecules with Identical Fingerprints

We provide additional groups of example compounds that share identical ECFP4[3] and MACCS[4] fingerprints yet exhibit significantly different acute dermal toxicity profiles, as exhibited in Supplementary Table S1 and S2.

Table S1: Example molecules from the rabbit dataset.

Group	SMILES	Toxicity
1	CCCCCCCO	ADT
	CCCCCCO	Non-ADT
	CCCCCCCCO	Non-ADT
	CCCCCCCCCO	Non-ADT
	CCCCCCCCCCO	Non-ADT
	CCCCCCCCCCCO	Non-ADT
	CCCCCCCCCCCCO	Non-ADT
	CCCCCCCCCCCCCO	Non-ADT
	CCCCCCCCCCCCCCO	Non-ADT
	CCCCCCCCCCCCCCCCO	Non-ADT
	CCCCCCCCCCCCCCCCCCO	Non-ADT
	CCCCCCCCCCCCCCCCCCCCO	Non-ADT
	CCCCCCCCCCCCCCCCCCCCCCO	Non-ADT
	CCCCCCCCCCCCCCCCCCCCCCCCO	Non-ADT
2	CCCCCCOCCOCCO	ADT
	CCCCCCCCCCCCCCOCCOCCOCCOCCOCCOCCOCCO	ADT
	CCCCCCCCCCCCCOCCOCCOCCOCCOCCOCCO	Non-ADT

Table S2: Example molecules from the rat dataset.

Group	SMILES	Toxicity
1	CCCCCCCCS	ADT
	CCCCCCS	Non-ADT
	CCCCCCCCCCCCS	Non-ADT
	CCCCCCCCCCCCCCCCCCS	Non-ADT
2	CCCCCCCCC(=O)O	ADT
	CCCCCCCCCC(=O)O	Non-ADT
	CCCCCCCCCCCCCCCCCCCCCC(=O)O	Non-ADT

S3. Visualization of Molecules in the Training Set

In this section, we apply t-SNE[5] to visualize the ECFP4 and MACCS fingerprints of molecules on the Rat and Rabbit datasets, as presented in Supplementary Figure S2.

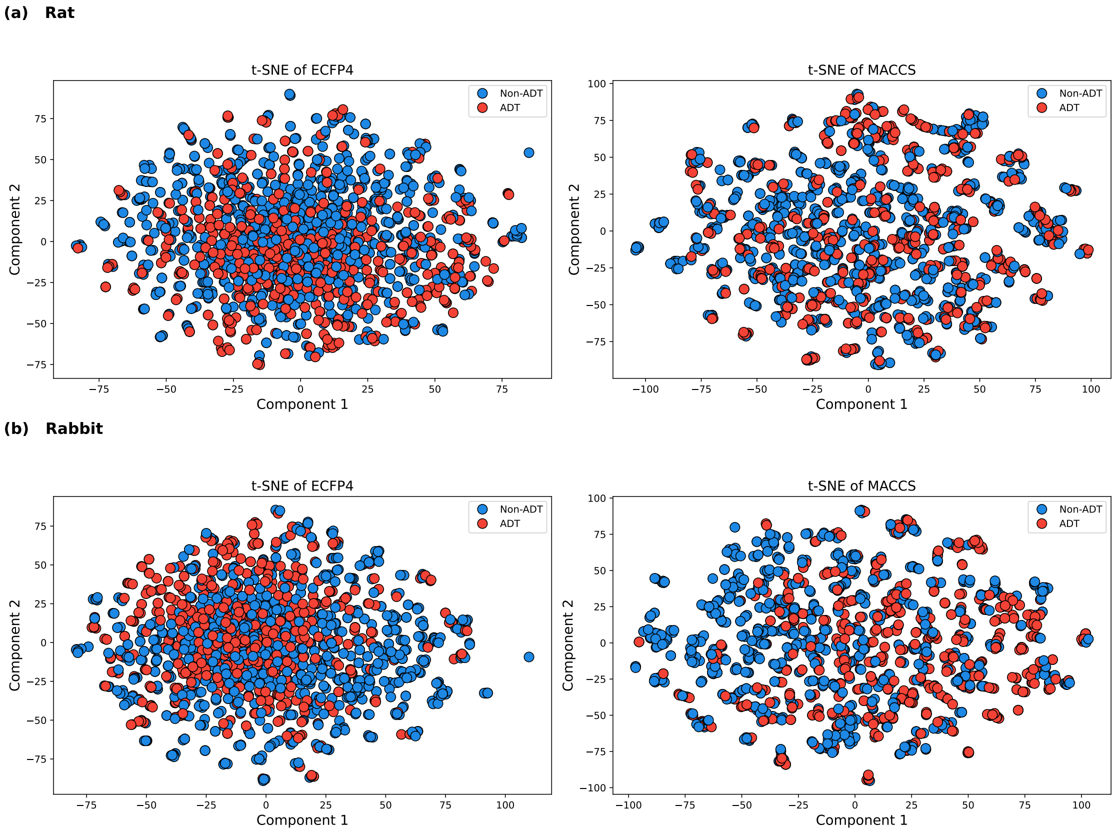

Figure S2: Plots of ECFP4 and MACCS molecular fingerprints on the training sets of (a) Rat and (b) Rabbit datasets visualized by t-SNE.

References

(1) Ramachandran, P.; Zoph, B.; Le, Q. V. Searching for activation functions. *arXiv preprint arXiv:1710.05941* **2017**,

(2) Glorot, X.; Bordes, A.; Bengio, Y. Deep sparse rectifier neural networks. Proceedings of the fourteenth international conference on artificial intelligence and statistics. 2011; pp 315–323.

(3) Rogers, D.; Hahn, M. Extended-connectivity fingerprints. *Journal of chemical information and modeling* **2010**, *50*, 742–754.

(4) Durant, J. L.; Leland, B. A.; Henry, D. R.; Nourse, J. G. Reoptimization of MDL keys for use in drug discovery. *Journal of chemical information and computer sciences* **2002**, *42*, 1273–1280.

(5) Maaten, L. v. d.; Hinton, G. Visualizing data using t-SNE. *Journal of machine learning research* **2008**, *9*, 2579–2605.
